# COGNITIVE ENHANCEMENT FOR GRANDPARENTING: REDUCED LONELINESS AS A MEDIATOR

**DOI:** 10.1093/geroni/igz038.1812

**Published:** 2019-11-08

**Authors:** Yuanqing Chang, Yin Li, Xin Zhang

**Affiliations:** 1 School of Psychological and Cognitive Sciences,Peking University, Beijing, China; 2 Peking University, Beijing, China

## Abstract

Grand-parental care, an important form of family care in China, However, its health effect on older adults, including their physical and mental health and cognitive function, is often not well understood. In the present study, we investigated the differences in terms of physical and mental health (measured by SF-36 Quality of Life Scale), self-aging attitudes (measured by the Philadelphia Senior Center Morale Scale), and cognitive functions (measured by WAIS, the subscales of Wechsler Adult Intelligence Scale) between those who provide grand-parental care and those who do not. Two studies were conducted. The first study examined 150 older adults who provided grandparental care and another 150 older adults who did not. Six months later, we randomly selected 103 older adults to conduct a longitudinal follow-up, of which 53 were older adults who provided grandparental care, and another 50 older adults were those who didn’t. The results of both cross-sectional and longitudinal studies showed that, compared with older adults who did not provide grandparental care, those providing grandparental care has significantly better physical and mental health, more positive self-aging attitudes and even enhanced cognitive functions. Further path analysis showed that loneliness mediated the association between providing grandparental care and enhancement in functions, such that providing grandparental care can reduce loneliness of the older adults, which in turn can improve their physical and mental health and even enhance their cognitive functions. These results shed light on the practical implications of grandparenting in China for the society.

